# A Case of Hay Fever

**Published:** 1884-12

**Authors:** W. S. Gee

**Affiliations:** Hyde Park, Ill.


### CLINICAL BUREAU.

### A CASE OF HAY FEVER.

A monograph on this subject appeared some years ago giving in a word Ars. iod. as the only remedy of special value, or, in fact, of any value, in this disease. Articles have lately appeared by two writers, both named MacKenzie. The result of these was nil as regards the management of the trouble. The Ars. iod. has not proven to be of value in but one case, so far as my experience goes. Ambrosia artimes. was given special mention a few years ago. This does seem to be of service in the asthmatic form *when indicated.* The value of a remedy in this, as in all other diseases, depends upon the similarity of the symptoms to those given by the patient.

This patient, a lady aged twenty-six, married, mother of two children, is slim and delicate; weight about eighty-five pounds; has had this distressing disease for ten years in succession; always had it very severely. Her life is made miserable by it.

September 4th, 1884.—She gave these symptoms as recorded then. The attack begins every year on August 15th to the very day. It comes with a nervous feeling, as though she would go wild, compelling her to walk about. Begins with sneezing in the early morning—"Sneezes twenty times," and seems as if it would tear her chest in pieces. Feels as though firecrackers exploded in right half of chest every time she sneezed. The eyes smart and burn; must rub them, which makes them worse; feel like something in them. After the paroxysms there is a small lump of cheesy, pus-like substance in the inner canthus of both eyes. Eyes feel blurred, objects are seen indistinctly, eyes feel weak. Eyes feel hot when first going into the open air but are better later. Water runs from the nose after sneezing; water is hot and burning, makes the nose sore. Then the throat begins burning and smarting, is dry, feels closed, so that she can't speak. There appear in the back of throat little white granular spots. Constant effort to clear the throat. The chest symptoms follow. There is a sore spot in right chest under the clavicle, about two inches in diameter, sore, burning, sensitive to touch, a feeling as though it had been burned. The cough is dry, worse from a draught of air; dreads the cold air, afraid of cold, yet feels relieved after she has been out a short time. Pain in the sore spot from coughing. No expectoration. Headache all the time, usually relieved after vomiting, but now vomits only water, and the headache continues; begins in the top of the head, extends to the left temple, is hot and throbbing; aggravated from any exertion; moving makes it very sore; hot applications give temporary relief. Appetite variable, fruit tastes best; thirsty, drinks much but not often; water does not quench her thirst, prefers beer. Has backache, with nausea at stomach, better when lying down. Leucorrhœa slight. During last flow (second day) felt as though everything would come out, as if bones of pelvis spread apart; flow too profuse and lasts too long. Aching in thighs from front to back; seems "deep in the bones." Sleep light and unrefreshing. Dreams of the affairs of the day. Palpitation on going up and down stairs or from exertion. Marked prostration. Perspires freely. Saliva runs from the mouth when asleep. Feels generally worse in the evening. Feels irritable and little things offend. Cal. carb.$^{mm}$

September 10th.—Took a powder the eve of the 4th and on the eve of the 5th, but felt so much better next day that she took the disks (Sac. lac.) alone. The first change noticed was that the "spells" were lighter in degree and length. Eyes have not troubled so much. Has not been so nervous, felt quiet. Not so sensitive to cold. Still has a choked feeling and the nose discharges *before* she sneezes. The former attacks *began* by "sneezing twenty times." There is less smarting and burning. Eyes feel stronger. Breathing better, does not have the tight feeling. The sensitive spot is comfortable except when sneezing. Throat more comfortable. Fewer little spots in the throat. Less of the closing feeling, "nearly gone." Feels much stronger. Has no need to hack or clear the throat. The spot is sore and slightly tender, but the burning is all gone. Coughs rarely. No dread of fresh, cold air. Went to a party Saturday (6th) and expected to be troubled greatly, but was entirely free from it. Headache absent since Friday (5th). Back "a great deal better." Has not noticed the salivation at night. S. L.

I have met the patient frequently since, and she said she had not a symptom of the trouble afterward. She has gained in weight remarkably fast since, and feels better than she has before in her life.

Symptoms prominent: takes cold very easily; the wind seemed to blow "right through her." Great weariness. Symp. 987 reads: "Sore pain in right mamma on the slightest touch." 990: "*During the cough pain in the chest as if raw*, evenings and nights." "Irritable without cause; peevishness and obstinacy." 340: "*Sore, ulcerated nostril, preceded by frequent sneezing.*" 348: "*Severe coryza, with headache* and oppression of the chest." Headache relieved by vomiting mucus and bile. C. H. G. 554: Great thirst for beer.

HYDE PARK, ILL. W. S. GEE.

